# A Practical and Theoretical Treatise on the Diagnosis, Pathology, and Treatment of Diseases of the Skin; Arranged According to a Natural System of Classification, and Preceded by an Outline of the Anatomy and Physiology of the Skin

**Published:** 1843-04

**Authors:** 


					Art. VII.
1.
AP radical and Theoretical Treatise on the Diagnosis, Pathology, and
Treatment of Diseases of the Skin ; arranged according to a natural
System of Classification, and preceded by an Outline of the Anatomy
and Physiology of the Skin.
By Erasmus Wilson, Consulting Sur-
geon to the St. Hancras Infirmary, Lecturer on Anatomy ana f nysio-
logy in the Middlesex Hospital School of Medicine, &c. &c ?London,
1842. 8vo, pp. 407.
2. Manual of Diseases of the Skin. From the French of MM.
Cazenave and Schedel. With Notes and Additions by Thomas H.
Burgess, m.d., Surgeon to the Blenheim-street Dispensary for Dis-
eases of the Skin, &c.?London, 1842. 12mo, pp. 320.
3. Nouvelle Dermaiologie, ou Precis theorique et pratique sur les
Maladies de la Peau, fonde sur une nouvelle Classification medicale,
avec un Formulaire special et Planches coloriees. Par P. Baumes,
Chirurgien en chef de l'Hospice de l'Antiquaille de Lyon, &c. &c.?
Paris, 1842. Deux Tomes, 8vo, pp. 560, 622.
New Dermatology ; or a Theoretical and Practical Treatise on Dis-
eases of the Skin, founded upon a new medical Classification, with
a special Formulary and coloured Plates. By P. Baumes.?Paris,
1842. Two Volumes.
Previous to the publication of Willan's works, several circumstances
1843.] on Diseases of the Skin. 373
combined to retard the study, and check the advance of our knowledge
of cutaneous diseases ; the principal of which were the obscurity and im-
perfections of the discriptions of the individual affections handed down
to us, and the disagreement in nomenclature and classification between
different writers upon the subject. The most opposite terms were often
employed to designate similar diseases; and the several stages of the same
affection were often described as disorders essentially different from one
another. To Willan, and his successor Bateman, the profession is in a
great measure indebted for the subsequent advances which have been
made, and for many of the improvements which have taken place in this
department of medical science. It must however be confessed that the
cultivation of cutaneous diseases as a distinct branch of pathology, has
in some measure languished in this country since the death of Bateman.
The introduction of the stethoscope, and the brilliant discoveries to which
it paved the way, seem of late years to have almost engrossed the atten-
tion of physicians, and to have drawn them away from the study of other
.important diseases. We hail, therefore, the almost simultaneous ap-
pearance of the two English works, the titles of which we have prefixed,
and the announcement by the journals of a third, as indications that
this portion of the field of medical science is beginning again to attract
the attention of the profession, and to take the position which its im-
portance demands.
The earliest attempts at a classification of cutaneous diseases were
necessarily very imperfect. In one of the most ancient they were arranged
according to their situation : one division had its seat upon the head, or
face; the other upon the body, or extremities. This classification was
partially adopted by Turner, one of the earliest English writers on the
subject, and was the groundwork of Alibert's first classification. Again,
cutaneous diseases were arranged according to the supposed cause pro-
ducing them, and two divisions were made as they depended upon ex-
ternal or local, and internal or constitutional causes. Subsequently they
were arranged in two classes, according as they had an acute or chronic
character; but as this mode of classification necessarily, in many cases,
separated the acute and chronic stages of the disease, and required them
to be treated of in separate sections, it never was generally adopted. The
only modern classification which approaches this in simplicity we had the
good fortune to learn in the days of our pupillage; its author (a prac-
titioner of the old school) never published upon the subject, and but for
the opportunity afforded us here, it would probably have been altogether
lost to posterity. Therapeutics formed the basis of this classification, and
in it cutaneous diseases were arranged in four orders or groups. The
first included diseases curable by sulphur. The second contained the
diseases curable by citrine or tar-ointment. The third included diseases
curable by Plummer's pill; and the fourth, diseases which could not be re-
ferred to any of the foregoing orders, and required more active medicines.
The first classification of cutaneous diseases, deserving the name of
scientific, was that of Professor Plenck, of the university of Buda, founded
upon the external appearances of the eruptions; which was soon succeeded
by the well-known one of Willan, founded upon nearly similar principles;
namely, the character which the eruptions present when perfectly and
fully developed. The object of this classification was to facilitate the
374 Wilson, Cazenave, Schedel, Burgess, Baumes, [April,
diagnosis of these diseases, and as it settled the nomenclature, and fixed
the meaning of the terms employed in designating them, it was calculated
.to lead to clearer views than any previous arrangement, and met with a
very favorable reception from the profession at the period of its publica-
tion ; indeed it was not only almost exclusively followed in this country,
but it received the nearly unanimous approval of our continental neigh-
bours, by whom it was characterized as the most methodical and accurate
of all the classifications of diseases of the skin hitherto proposed.
Since the death of Bateman, the profession in this country (with one
or two exceptions) seems to have been content to follow in his footsteps,
considering that the Willanean classification was as perfect as the nature
of the subject permitted. In France, too, the system of Willan, with
slight modifications, was adopted by the most distinguished teachers and
writers; as, for instance, Biett, Rayer, Gibert, Cazenave and Schedel.
Alibert, however, must be excepted; who, at different periods, proposed
and taught two distinct forms of classification : but his systems (as Mr.
Wilson observes) live at the present day only in the memory of the past,
and have ceased to exist even beneath the foliage of the ' tilleuls that
smiled upon their birth. Mr. Plumbe, in this country, was the first syste-
matic writer who called in question the classification of Willan, and proposed
a different one ; " the basis of his arrangement being founded on the con-
stitutional causesof the disease, and due consideration oftheorganicstruc-
ture and physiology of the part of the skin on which it is seated." There
is, unquestionably, (as Rayer observes,) considerable ingenuity in several
parts of Mr. Plumbe's classification, but it is inferior to that of Willan,
and it never was generally adopted in this country.
The classification followed in the Manual of MM. Cazenave and Schedel
(so well translated by Dr. Burgess) is that of Willan, modified and im-
proved by M. Biett. In this method, cutaneous diseases are arranged in
fifteen orders; the first eight, or principal orders, contain the great ma-
jority of cutaneous diseases. The following table, given at page 5, con-
tains a view of this arrangement.
" Order 1. Exanthemata.?Erythema, erysipelas, roseola, rubeola, scarlatina,
urticaria.
" Order 2. Vesiculce.?Miliaria, varicella, eczema, herpes, scabies.
"Order 3. Bulla;.?Pemphigus, rupia.
" Order 4. Pustulce.?Variola, vaccinia, ecthyma, impetigo, acne, mentagra,
porrigo, equinia or glanders.
" Order 5. Papules.?Lichen, prurigo.
" Order 6. Squama.?Lepra, psoriasis, pityriasis, ichthyosis.
" Order }. Tubercula ?Elephantiasis Grecorum, molluscum, frambaesia.
"Order 8. Rlaculce, Colorationes.?Fuscedo cutis, ephelides, nsevi. Be-
colorationes.?Albinismus, vitiligo.
"Order 9. Lupus. 10. Pellagra. 11. Malum Alepporum. 12. Syphilida.
13. Purpura. 14. Elephantiasis Arabica. 15. Cheloidea."
This arrangement agrees, in most respects, with that of Willan : the
first eight orders are similar to his, and differ only in the position of some
of the genera. For instance, erysipelas has been very properly removed
from the order Bullae; scabies is a vesicular, not a pustular disease;
acne and sycosis, which Willan classed with tubercular diseases, have been
placed in the order Pustulse ; pemphigus and pompholyx are considered
to be varieties of the same disease, and are both described under the term
1843.] on Diseases of the Skin. 375
pemphigus; purpura, classed with exanthemata (with which it has no
analogy), and lupus with tubercle, by Willan, have been both placed in
orders by themselves.
Willan's classification has been called artificial, because it is founded
upon a single character, (viz., the appearances presented by the diseases
when at their full development,) and because neither the etiology nor the
tissue affected are taken into account as elements of the arrangement.
" In this respect it corresponds very aptly," Mr. Wilson observes, " with the
Linnaean system of classification in the vegetable kingdom, which is like-
wise designated artificial. But while the Linnaean system is admitted,
on all hands, to be that by the aid of which a tyro may most certainly
and expeditiously discover the name of a flower, and then, by referring
to his books, learn its history and properties, the system has fallen into
disrepute among skilful botanists, and has given place to one founded on
a more philosophical basis, which is called the natural system." (p. viii.)
While examining the advantages and disadvantages of Willan's arrange-
ment, and comparing with it the classification applied to botanical science,
the idea occurred to Mr. Wilson that a natural system of arrangement
might be applied to diseases of the skin ; he followed up this idea, and the
result has been the production of a classification, which he terms, " A
Natural System of Diseases of the Skin." The basis of this classification
" rests upon anatomy and physiology The dermis and its appen-
dages, its glands and follicles, are considered to be the seat of all the
changes which characterize cutaneous pathology." These, therefore, con-
stitute the four primary divisions; and the following tabular view, con-
tained at p. xxv, gives an outline of his classification.
s
Inflammation
Diseases of the Dermis.
- Rubeola.
I Scarlatina.
Specific  ^ Variola.
Varicella.
Congestive  ^ v Vaccinia.
Erysipelas.
-T .c , Urticaria.
?Non-specific  } Roseola.
C. Erythema.
J Asthenic  {
Effusive   \ r Herpes.
V Sthenic   ' Eczema.
Sudamina.
Impetigo.
Ecthyma.
Suppurative   |
C Strophulus.
Depositive   ^ Lichen.
(_ Prurigo.
C Lepra.
Squamous   < Psoriasis.
(__ Pityriasis.
v From Parasitic Animalcules   Scabies.
, Icthyosis.
\ Tylosis.
Hypertrophy of the Papillse   -s Clavus.
J Verrucae.
^ Cornua.
376 Wilson, Cazenave, Sciiedel, Burgess, Baumes, [April,
Disorders of the Vascular System   | Purpura' n3eV'"
Disordered Sensibility   ^ Prur^tus^68'8"
Augmentation of Pigment   { Pigmentary
Diminution of Pigment    ^
Disordered
Chromatogenous
function
{
naevi.
Albinismus.
Vitiligo.
Ephelis.
Lentigo.
Alteration of Pigment  ^ Chloasma.
Melesma.
. , r, , S Oxide of Silver
^Chemical Coloration   | gtain
II. Diseases of the Sudoriparous Glands.
Augmentation of Secretion   Sudatoria.
Diminution of Secretion   ) Abnormal odour, colour, &c.
Alteration of Secretion   S
III. Diseases of the Sebaceous Glands.
Augmentation of Secretion    Stearrhoea.
Diminution of Secretion   J Icthyosis sebacea.
Alteration of Secretion   J
t C Comedones.
) Sebaceous accumulations.
Duct ?Pen  ) Small sebaceous tumours.
Retention of Secretion / ' (Molluscum contagiosum.)
C Sebaceous miliary tubercles.
Duct closed  \ Calcareous miliary tubercles.
l Serous cysts.
^ V. Encysted tumours.
Inflammation of Glands and adjacent Textures... | Sycosis.
IV. Diseases of the Hair and Hair Follicles.
Augmented Formation    Pilous naevi.
Diminished formation    ? CaMtfeT.'
Alteration of Colour  Canities.
Disease of the Hair-pulps   Plica Polonica.
, ,, ? , ( Inflammatio folliculorum.
Disease of the Follicles   i Favus
f Trichiasis.
Abnormal Direction   ? Felting.
M. Beaum&s employs two kinds of classification for cutaneous diseases;
one he calls the " Dermatological," the other the " Dermatographical;"
the principal novelty in hismethod belongs to the former. Thus,he considers
all cutaneous eruptions to be the result of a morbid condition, which he
terms congestion, "fluxionand he believes the different forms which
they assume may be traced to a definite number of distinct causes; upon
these assumed causes his dermatological classification is based. He has
made seven classes of cutaneous diseases, and the following is a summary
of his arrangement.
The first class includes the cutaneous eruptions which depend upon
" congestion from an external causeas, for instance, when the matter
of a contagious disease (such as the itch) gives origin to a similar disease
1843.] on Diseases of the Skin. 377
in the part to which it is applied, without the constitution, being primarily
affected.
The second class includes diseases which are symptomatic of, and ap-
pear to depend on, some internal disease, in which case the eruption is
said to be from " reflected congestion."
In the third class, a cutaneous eruption supplies the place of a sup-
pressed evacuation, or of some morbid process in another tissue, when the
disease is said to be from " misplaced congestion."
The fourth class includes diseases which are the result of some cause
acting upon the whole system, either through the nerves or blood; as
excesses of all kinds, deficiency of food, strong mental emotions, &c.;
these he terms cutaneous eruptions from " eccentric congestion."
The fifth class contains cutaneous affections which arise from some pe-
culiarity in the constitution or diathesis of the patient, (as the scrofulous,
cancerous,) in which case the eruption depends upon " congestion from
diathesis."
The cutaneous diseases placed in the sixth class are developed spon-
taneously, or without any appreciable cause, when the eruption is said
to be by " idiopathic congestion."
In the seventh class, several of the foregoing causes may combine to
produce the disease, in which case the eruption depends on " complex
congestion."
In M.Baum&s' "dermatographical" classification, diseases are arranged
in eight orders; viz., 1, Erythematous eruptions; 2, Vesicular, or
pustulo-vesicular; 3, Papular; 4, Tubercular; 5, Scaly, or furfura-
ceous eruptions; 6, Maculae; 7, Excrescences, vegetations, and cu-
taneous tumours; 8, Diseases, or alterations of the appendages of the
skin. As there are a certain number of diseases which do not admit of
being included under any of the foregoing orders, he has placed them in
a section apart; viz., erysipelas, urticaria, acne, sycosis, lupus, &c.
M. Baum&s then subdivides the orders according to the cause which he
supposes to produce each disease contained in it; but the inapplicability
of this method to all cases is proved in his work, as the author has found
it necessary to describe the most important diseases belonging to each
order, not under the heads of his " dermatological arrangement," but in
separate and distinct sections.
In regard to these different forms of classification, we must confess that
old associations and early prepossessions on our side, are linked with
Willan's arrangement: like the Linnsean classification of the vegetable
kingdom, it is the one with which we commenced the study of the subject,
and it has become almost a habit to follow and be guided by it. One of
the principal ends of a classification of cutaneous diseases is to enable the
practitioner, or student, to discover the object of his search in the readiest
manner possible, and with the least trouble; the Linnsean classification
of the vegetable kingdom leads to the expeditious discovery of the name
of the plant sought, after which other sources, for further information, can
be referred to. If the Willanean arrangement of cutaneous diseases pos-
sesses this advantage it certainly is a great one, and it should not be set
aside on light grounds; that it does so is admitted by Mr. Wilson, who
observes (p. viii), "none can be better suited (than the Willanean) to
lead the student to the discovery of the name of the disease, when the
378 Wilson, Cazenave, Sciiedel, Buiigess, Bauaies, [April,
disease is at its .height; but if he be called to study it at the rise or the
decline, the system of Willan, like the Linnsean, is but a faithless and
deceptive guide." The analogy between the Linnsean classification and
that of Willan does not, however, appear to us to be so close as Mr. Wilson
would lead the reader to suppose. In the botanical arrangement a diffi-
culty no doubt arises in many cases, on the fall of the stamina, or before
the development of the flower; but in a large number of cutaneous dis-
eases, the characters upon which the eruption is classified may be detected
(if carefully looked for) in every stage of the disease. With every dispo-
sition, therefore, to give Mr. Wilson credit no less for the ingenuity with
which he has worked out the details of his classification, than for the talent
which he has brought to bear upon the whole subject; and admitting that
his is the most scientific, and most natural arrangement, we must again
repeat, that the principal object of a classification of cutaneous diseases
being to facilitate the recognition of the species, and to lead to the ready
detection of the disease, an arrangement which fulfils these conditions
must continue to be the most popular with the student and practitioner:
and so far as classification, this condition is fulfilled by Willan's system,
as modified by Biett, Cazenave and Schedel, and adopted in the Manual
of Cutaneous Diseases by the latter gentleman, translated by Dr. Burgess.
We think, therefore, that in practice Dr. Burgess's volume will be found
to obtain the preference. We wish, however, not to be misunderstood
upon this point; we are quite aware that there are positive defects in the
Willanean system. From its artificial nature it must sometimes associate
diseases of opposite characters, and must also separate others which are
intimately allied (defects from which Mr. Wilson's arrangement is free);
but it appears to possess these advantages over his,?that it is more sim-
ple ; the orders under which the diseases are arranged are less numerous;
and the characters by which they are distinguished from each other are
consequently more readily remembered.
M. Baumes' system (founded upon the etiology of cutaneous diseases)
need not detain us long; it appears to be based upon the views of Lorry
proposed so long ago as the year 1777. In some points, also, it bears
an analogy to the arrangement adopted by Mr. Plutnbe. Almost all
writers on the subject, however, appear to be agreed that the etiology of
diseases of the skin is far too obscure ever to serve as the basis for their
arrangement. Indeed, M. Baumes seems (as Rayer has remarked) to
have overlooked the advantages of the Willanean arrangement, to have
erroneously disputed the utility of determining species, and gratuitously
to have supposed that those who adopted Willan's system took no notice
of the other characters of the diseases of the skin in establishing their
diagnostic features. " His classification (in the words of M. Gibert)
pays little attention to the characters of cutaneous diseases; finding in
congestion, revulsion, and sympathy, an explanation of all the morbid
phenomena comprised in cutaneous pathology, and is calculated to throw
us back into the chaos from which modern observers have extricated us."
Anatomy and Physiology of the Skin. The first chapter of Mr.
Wilson's work is devoted to the anatomy and physiology of the skin ;
and as these subjects are not noticed in either Dr. Burgess's Manual, or
M. Baum&s' larger work, we shall confine our observations on this head,
to an abstract of some of the details contained in Mr. Wilson's volume.
1843.] on Diseases of the Skin. 379
The skin, from the days of Malpighi down to a very recent period, was
supposed to consist of three distinct layers, viz. the cuticle or epidermis,
the rete mucosum, and the cutis, dermis or true skin. The result of the
researches of MM. Breschet and Roussei de Vauzeme led them to con-
sider the skin as composed of six constituent parts, viz., the dermis, pa-
pillae, perspiratory apparatus, apparatus of inhalation or absorbent canals,
apparatus producing the mucous matter, and an apparatus producing the
colouring matter. The skin is now usually believed to consist of two
layers, the "epidermis and dermis ; with secretory organs, the sudoriparous
and sebaceous glands; and of certain appendages, as the epidermis, the
hair and nails. The epidermis and dermis are each composed of two
layers : the superficial layer of the former is the cuticle ; the deeper layer
the rete mucosum of the old writers; the superficial layer of the dermis is
named the papillary, the superstratum, the corium.
The dermis, or cutis, varies in thickness in different parts of the surface :
on the back, outer sides of the limbs, palms of the hands and soles of the
feet, it has a greater density than upon the inner surface of the limbs,
the eyelids, or scrotum, &c. It is composed of areolo-fibrous tissue,
elastic and contractile fibrous tissue, nerves, blood-vessels, and lympha-
tics. The superficial portion of the areolo-fibrous tissue supports the
papillae, and is formed of an intricate interlacement of white fibres, col-
lected into small fasciculi; its deeper layer is composed of broader fas-
ciculi, which leave interspaces in which small masses of adipose tissue
are lodged. The contractile fibrous tissue of the dermis is composed of
fibres sometimes collected into fasciculi, sometimes interlacing; they are
most abundant in the coarse network of the under surface of the corium;
this structure is more readily seen in the corium of animals than in the
human subject,?in the latter it has only been demonstrated in the nipples
and scrotum; the appearance of the skin produced by cold, termed cutis
anserina, is owing (Mr. Wilson observes) to the presence of this con-
tractile fibrous tissue.
The papillary layer of the dermis consists of conical minute promi-
nences, which upon the surface generally are exceedingly minute and
very irregular in their distribution, but in some situations as the palm of
the hand and sole of the foot are larger, and are arranged in a regular
order ; on these parts the papillae are collected into little square masses,
each containing from ten to twenty, which are disposed in parallel rows ;
and it is this arrangement of the papillae which gives rise to the parallel
ridges and furrows seen upon the palm of the hand and sole of the foot.
The largest papillae are those which produce the nail, and their form and
arrangement are peculiar. Near the root of the nail, and for a short
distance onwards the papillae are minute and less vascular than those
further on. "This patch of papillae is bounded by a semilunar line of which
the concavity is turned towards the root, and in consequence of appearing
lighter in colour than the rest of the nail, it has been termed the cu?iula."
The epidermis is a secretion of the dermis, which it serves to protect;
its superficial layer is dense and unorganized, its inner layer (rete muco-
sum,) is soft and cellular, and in contact with the papillary layer of the
dermis. The cuticle is laminated in its texture, and as the superficial
laminae are continually removed by attrition, new laminae are continually
reproduced upon its internal surface. In situations where the cuticle is
380 Wilson, Cazenave, Schedel, Burgess, Baumes, [April,
much exposed to pressure, as the soles of the feet, it is thick ; in others it
forms a very thin layer, and " its degree of thickness is dependent on,
and bears an accurate proportion to the degree of development of the
papillse of the dermis. The epidermis is accurately modelled on the
papillary layer of the dermis, and each papilla has its appropriate sheath
in the newly-formed epidermis or rete mucosum." (p. 6.) The different
tints of colour of the skin observable in different nations, depend upon
the presence of pigment in the cells of the epidermis.
The sudoriparous glands are seated in the subcutaneous areolar tissue;
they are small oblong bodies, in some cases sacs, in others tubuli, ending
in minute spiral tubes, called perspiratory ducts, which pass through the
dermis and epidermis, and terminate by a pore upon the surface of the
latter; they are lined by an inflection of the epidermis; the pores are
readily seen upon the palmar surface of the hands.
The sebaceous glands, which are sometimes simple pouch-like follicles,
at others lobulated glands, are seated in the substance of the dermis; their
excretory ducts generally terminate in a hair follicle, and are lined by a
reflexion of the epidermis ; they occur on every part of the surface, are
numerous on the scalp, face, arm-pits, and perineum ; in the meatus
auditorius they are large, and have received the name of ceruminous
glands; the meibomian glands are the largest in the body. They secrete
an oily fluid which preserves the flexibility of the cuticle, and protects
parts much exposed to friction.
" In a physiological point of view the skin is an organ of sensation, absorp-
tion, and secretion ; in the former capacity it affords us gratification, and warns
us of the presence of injurious or destructive agents; by means of the second it
is enabled to appropriate the fluids contained in the surrounding medium, and
perform the office of a respiratory organ; and by means of the third, it provides
for its own softness and pliancy, it regulates the influence of temperature both
external and internal, and acts as an important depurating organ of the blood."
(p. 14.)
Congestive Inflammation of the Dermis. Under this title Mr.
Wilson has assembled a group of diseases, the leading feature of which
is inflammation, and consequent redness of the skin. He then makes two
sub-groups : the first including such as are characterized by " inflam-
mation of the dermis and mucous membrane, with constitutional symp-
toms of a specific kind," namely, rubeola, scarlatina, variola, varicella,
and vaccinia ; the second such as are characterized by " inflammation of
the dermis without constitutional symptoms of a specjiic kind," namely,
erysipelas, urticaria, roseola, and erythema. This class corresponds in
some respects with the order exanthemata of Willan, and with the divi-
sion ' eruptions erythemateuses' of M. Baum&s; it differs in including
variola, vaccinia, and varicella, which have been classed among vesicular
and pustular diseases by those authors. The diseases placed in the first
sub-group, constitute pretty nearly Cullen's order exanthemata, whose
well-known definition fully expresses their characters, viz. " contagious
diseases, attacking a person only once in his life, beginning with fever;
at a definite period eruptions appear, often numerous, small, and scat-
tered over the skin." The cutaneous eruption in these diseases is pre-
ceded by the fever, (as expressed in Cullen's definition,) and the consti-
tutional affection is not the consequence of the eruption; hence several
1843.] on Diseases of the Skin. 381
writers look upon the exanthemata as more properly belonging to diseases
of the system than to the division of cutaneous diseases, and upon good
grounds, when we find a variety of measles, and one of scarlatina, cha-
racterized by being unaccompanied by any cutaneous eruption, as the
rubeola and scarlatina, sine exanthemate, and for these reasons both M.
Gibert and Mr. Plumbe have omitted altogether the consideration of
rubeola, scarlatina, variola, and erysipelas in their works on diseases of
the skin.
The seat of the inflammatory congestion in the exanthemata, according
to Mr. Wilson, " is the vascular rete of the dermisin rubeola, scarlatina,
and variola, many of the papillae of the dermis are also " distended with
blood, producing the punctiform appearance of the redness which is cha-
racteristic of them and the crescentic form of the congested patches in
rubeola depend upon " some unexplained peculiarity in the structure of
the dermis, probably having reference to the distribution of the cutaneous
nerves." The mottled aspect of the skin of healthy children exposed to
'cold has the same semilunar tracery; Mr. Wilson observes that, in
injecting the limb of an infant with size and vermilion, he could imitate
all the forms of redness seen in exanthematous diseases, by ceasing to
inject, from time to time ; or by filling the capillaries to their utmost.
The diseases included in the first sub-group, being infectious and con-
tagious, it may be well (Mr. Wilson remarks,) to inquire the precise
meaning which we attach to these terms :
"When the transmission is effected by a material substance, and is brought
about by actual contact, the term contagion (immediate contagion,) is employed;
but when transmission is effected through the agency of tne winds, and at a
distance, the mode of communication is designated infection (mediate con-
tagion). In other words, when the poisonous principle is volatile, and capable
of diffusion in the atmosphere, it is infectious; but when this diffusibility is ab-
sent, it is simply contagious. In whatever way the poisonous principle be brought
to the body of a sound person, and with whatever part it may come in contact,
whether with the cutaneous surface with or without abrasion, as in con-
tagion, or with both the cutaneous anil mucous surface as in infection, the mode
of its reception by the system is the same. In the first instance, it is dis-
solved in the fluids of the body, and in the second place, is conveyed by imbi-
bition into the circulating current of the blood, thence to act on the nervous
system, and alter its functions. Once introduced, the poisonous principle pos-
sesses the remarkable power of exciting an action similar to that which existed
in the body whence it emanated, the intention of that action being the repro-
duction of an identical poison." (p. 25.)
Liebig, as Mr. Wilson remarks, has compared the phenomena which
succeed the introduction of an animal poison into the blood, to the pro-
cess of fermentation ; for instance, when a little yeast is added to a fer-
mentable fluid, the action which it excites occasions the formation of
abundance of similar yeast; so the poisonous principle on reaching the
blood reacts upon it, and causes the formation of the poison from the
constituents of the blood. A remarkable phenomenon connected with
these diseases is that they in general occur but once in a person's life,
and having once occurred, the system is protected in a great measure
against a similar attack. Liebig's theory alone offers any explanation of
this curious fact, viz., that we cannot, in a fluid, which has already fer-
mented, excite a fresh fermentation of the same nature ; " it is upon this
VOL. XV. NO. XXX. *7
382 Wilson. Cazenave, Schedel, Burgess, Baumi?s, [April,
principle that safety from a repetition of attacks of eruptive fever reposes,"
Another point connected with these diseases deserving of remark, is
that from the time at which the contagion has been applied, until the
disease begins to develop itself, a certain period intervenes (the period
of incubation,-) which varies in the several diseases belonging to this
group: thus in variola, according to Dr. G. Gregory, the period of latency
is usually about twelve days; in measles, according to Bateman, from
ten to fifteen days ; and in scarlatina from four to six days.
We cannot be expected to follow the authors through the characters,
symptoms, and diagnosis, of the several varieties of measles and scarla-
tina, we must be satisfied with giving the following summary and table of
the diagnostic characters of the two affections from Mr. Wilson's work :
Scarlatina.
1. Precursory symptoms of one days'
duration.
2. Mucous membranes of the eyes,
nose, and fauces red and inflamed,
without secretion: pain and soreness
of throat, no cough ; no expectoration.
3. Eruption on the second day of
the fever ; invades the entire surface of
the body in three days ; disappears by
the end of the seventh day.
4. The efflorescence occurs in large
irregular patches, or is more or less
generally diffused ; is of a bright scar-
let, compared by Willan to a "boiled
lobster," and frequently interspersed
with numerous small red papillae.
5. Odour resembling old cheese.
6. Principal sequelae, anasarca, in-
flammation of the joints, gangrene,
chronic bronchitis, ulceration of fauces,
conjunctivitis, otitis, abscess of salivary
glands, chronic diarrhoea.
7- Exfoliation of the epidermis in
laminae.
8. Less infectious and contagious
than measles.
9. Rarely attacks the same person
more than once.
Rubeola.
1. Precursory symptoms of three
days" duration.
2. Mucous membrane of the eyes,
nose, and fauces red and inflamed, with
increased secretion, coryza, sneezing,
&c., dry cough at first, subsequently
expectoration.
3. Eruption on the fourth day of the
fever, occupies three days in invading
the entire surface of the body; dis-
appears by the end of the eighth day.
4. The efflorescence occurs in small
crescentic, and circular patches with
intervening unaffected portions of the
skin ; the colour is darker than in scar-
latina, " with nearly the hue of a rasp-
berry," andinterspersed with numerous
small red papulae, disposed in clusters.
5. Odour sweetish, until the decline
of the eruption, then sourish.
6. Principal sequelae. The same as
scarlatina, with the exception of ana-
sarca, inflammation of the joints and
gangrene.
7. Exfoliation of the epidermis in
furfuraceous scales.
8. More infectious and contagious
than scarlatina.
9. Frequently attacks the same
person twice.
Treatment. In the milder forms of measles and scarlatina, the treat-
ment should be simply expectant; the disease is the result of the ab-
sorption of a morbid poison ; it has a certain course to run, and the less
the patient is interfered with by the employment of medicine the better;
Sydenham indeed remarked of scarlatina " that none die of this disease,
except from a too great officiousness on the part of the practitioner."
The diet should be spare, diluents freely employed, the apartments ought
to be kept cool and of an uniform temperature ; and there can be no objec-
tion to the administration of an emetic of ipecacuanha at the commence-
ment, nor to occasional laxatives of a mild kind (if required,) during its
1843.] on Diseases of the Skin. 383
progress; but the frequent employment of purgatives, or emetics, doses
of tartar emetic, (a practice but too common,) " is supported," as Bateman
remarks, "neither byexperience or principle,and is verylikely todoharm."
In the more severe forms of these diseases, or when they are complicated
with inflammation of internal organs, the treatment must be conducted
on general principles; venesection is sometimes necessary, local bleed-
ing more frequently; saline aperients may be employed, but the frequent
employment of active purgatives will prove more injurious in this than in
the milder forms of the diseases.
Belladonna in small doses was recommended by Hahnemann on homoeo-
pathic principles as a prophylactic against scarlatina; we have had no ex-
perience of its property in this respect. Dr. Sims (quoted by Mr. Wilson)
remarks, in relation to prophylactic treatment, " the best preventive to
the disease I found to be rhubarb taken in the quantity of a few grains
every morning so as to produce one laxative motion in the day. I did
not see one who used it confined afterwards to bed, though several
persons began it after they were infected, but before the period of their
sickening." MM. Cazenave and Schedel observe that sulphuret of
antimony and calomel in combination have been employed (with a
similar view) with advantage in the dose of one sixteenth to one eighteenth
of a grain of each for a child from two to four years old, repeated several
times a day. For protection against the spreading of the contagion of
scarlatina or measles, patients recovering from either ought to be se-
cluded from three weeks to a month.
Variola. Treatment to prevent pitting. We can only afford room
here to notice some of the local means which have been employed to
diminish the chance of cicatrices and marks being left by smallpox.
This point in the treatment is dwelt upon at some length by Mr. Wilson,
but is hardly noticed in Dr. Burgess's Manual, and the practice is dis-
couraged by M. Baum&s. To relieve the itching of the face during the
suppurating stage various emollient decoctions have been employed.
Mr. Wilson recommends the face " to be frequently bathed with an in-
fusion of mallows, or poppy heads, or weak barley-water, and the secre-
tions to be removed by means of a sponge wet with any of these fluids."
We prefer as a lotion a very dilute solution of chloride of soda; and the
sufferings of the patient are often mitigated by the frequent application
(by means of a feather) of the carron oil, long known as a valuable ap-
plication in recent scalds and burns. In all cases it is essential to pre-
vent the patient from scratching the pustules. It was the practice for-
merly to open the pustules to squeeze out the pus, and bathe and cleanse
the surface with tepid milk and water or some emollient decoction; this
plan M. Rayer has always found to be beneficial, and Mr. Wilson con-
siders that it accelerates very materially the healing of the ulcerations,
and prevents the formation of deep and disfiguring cicatrices.
It was the practice when smallpox was much more common than it is
at present, to exclude the light from the apartment occupied by the
patient in order to prevent pitting; the idea seems to have arisen from
observing the fact that cicatrices and marks usually occur only upon the
face, and part of the surface constantly exposed to the light. So recently
as 1832, Dr. Picton of New Orleans (quoted by Mr. Wilson) asserted
that in his practice no instance of pitting after smallpox occurred when
384 Wilson, Cazenave, Sciiedel, Burgess, Baum^s, f [April,
the light was shut out. In a discussion at the French Academy of
Sciences, (July 4th, 1842,) on the influence of light on the smallpox
pustules, M. Serres stated that he had made numerous experiments by
covering the pustules with small glass cups, and observed that they were
developed, modified in their progress, or completely arrested, according
to the degree of transparency of the glass. The same author has re-
marked (quoted by Mr. Wilson) that he never reaped such successful
results in the cure of smallpox as he did at La Pitie during one year,
that the patients were placed in a kind of cellar, which was very dark
and ill ventilated.
In order to cut short the progress of the eruption, or to arrest its de-
velopment during the papular or vesicular stage, various ectrotic (from
eKrirpwtTKw, to miscarry or render abortive) methods have been employed.
MM. Velpeau and Bretonneau proposed cauterization with the nitrate of
silver; the apex of the pustules being removed, a sharp pencil of nitrate
of silver was inserted into each, or a strong solution introduced by
means of a probe; these proceedings give so much pain and are so ex-
ceedingly tedious that they cannot often be employed. A solution of
nitrate of silver containing a scruple to the ounce of water, and applied
over the surface by means of a camel's hair pencil has been sometimes
substituted. The application of the solid nitrate of silver is more
effectual unless when the solution is employed very early, and even then it
scarcely ever checks the progress of the eruption ; an ointment composed
of two drachms of sulphur to the ounce of lard, applied several times a
day by means of slight friction, at an early stage of the disease, has been
recommended by Dr. Midivaine, of Ghent, as an ectrotic agent; it has
never been tried in this country. More recently mercurial ointments or
a plaster containing mercury, as the emplastrum vigo cum mercurio of
the French pharmacopoeia,* have been strongly recommended. They
should be applied on the second or at the latest on the third day of the
eruption ; the whole face is covered with a mask of the plaster, leaving
merely a space for the eyes, nostrils, and mouth, or the surface is co-
vered with a layer of the ointment: both are allowed to remain on for
three or four days. "The effect," says Mr. Wilson, " is to produce im-
mediate resolution of the eruption, or to arrest it at the papular or vesi-
cular stage ; it never becomes purulent, and the skin between the pustules
is never inflamed or swollen." According to M. Briquet mercury
possesses precisely the same influence over vaccinia as over variola.
Vaccination. Vaccination, although so simple an operation, is not
always performed with sufficient care, nor are the conditions requisite
for its success always sufficiently attended to; we cannot, however, find
space for Mr. Wilson's valuable observations upon this subject, but must
refer the reader to the work itself.
Effusive inflammation of the dermis. Under this heading Mr.
Wilson considers the cutaneous eruptions, especially characterized by
effusion of a serous fluid upon the surface of the dermis, and the con-
sequent elevation of the epidermis in the form of vesicles or blebs. It in-
cludes the orders bullae and vesiculse of Willan, Rayer, Cazenave and
* This plaster consists of the following ingredients: 95 mercury; 48 balsam of storax ;
318 common plaster; 16 of wax, resin, and turpentine, each ; 5 of ammoniac, bdellium,
olibanum and myrrh, each ; 3 saffron ; 2 spirit of lavender.
1843.] on Diseases of the Skin. 385
Schedel, and appears to be a natural arrangement of these groups ; in-
deed, in the original sketch of his classification, Willan had combined
those two orders together under one heading, but he was afterwards in-
duced to separate them. Mr. Wilson makes two divisions of this class:
one the asthenic, "characterized by diminution of the vital powers of the
system the second or sthenic, " marked by increased energy of the
nervous and vascular systems." The former comprises pemphigus and
rupia, the bullae of other writers ; the latter contains herpes, eczema, and
sudamina, the vesiculae of other writers.
Bullce are " elevations of the epidermis caused by the effusion of serum
or a sero-purulent fluid under itthey have received several names, as
blebs, blisters, phlyctense, and vary in size from that of a split pea to
an orange, "their shape is usually rounded;" they sometimes assume an
acute form, but are more frequently chronic ; and may appear upon any
part of the surface, or pass from one part to another. The bullae are
small at first, but usually attain a considerable size in twenty-four hours;
" at first they are also tense, but when the fluid thickens they become
flaccid as if only partially filledafter a time they burst, and are suc-
ceeded by " excoriations, incrustations, or ulcerations." Their diagnosis
is easy when the disease is at its height, as vesiculae are always much
smaller, and more numerous; " but when the bullae have burst, and are
succeeded by crusts, the diagnosis becomes one of more difficulty."
Pemphigus. Both Mr. Wilson and MM. Cazenave and Schedel follow
Rayer in regarding the several species of pompholyx as varieties of pem-
phigus; and in making two principal subdivisions of the disease, viz.
into acute and chronic. The acute form of pemphigus is a rare disease,
principally attacks children, is usually mild and of short duration, and
requires but simple treatment; the pompholyx solitarius and benignus,
and the pemphigus vulgaris of Willan are only varieties of it. Chronic
pemphigus (pompholyx diutinus of Willan) is a more frequent and
severe affection, occurs usually in old persons whose constitutions are
dibilitated or broken down, and is most common in the male ; its ap-
pearance is not preceded by fever ; it differs also from the acute form of
the disease in the mode of development of the bullae, which is always
successive, and in its long continuance which may be for months. The
structural change in the skin which occurs in pemphigus is precisely
similar to that which results from the application of blistering plaster, or
boiling water; indeed, Rayer has related a case where the disease was
simulated by the application of powder of cantharides.
The causes of pemphigus are not known in some cases, it has appeared
to be congenital, in others to be endemic or epidemic ; the acute form
usually occurs during the summer, and has been apparently excited by
the irritation of teething, excess or change of food, &c. The chronic
form occurs, as before observed, in individuals whose constitutions are
injured by intemperance or other causes, sometimes after continued
fatigue and anxiety, with poor and scanty food; " not unfrequently
(according to Bateman,) it is connected with anasarca, scurvy, purpura,
and other states in which the powers of cutaneous circulation are feeble."
Mr. Wilson has seen the disease as a sequela of scarlatina, it is some-
times complicated with prurigo or herpes, more frequently with chronic
affections of the viscera; the condition of the liver known under the name
386 Wilson, Cazenave, Schedel, Burgess, Baumes, [April,
of fatty liver has been found in individuals dying of this disease. M.
Rayer mentions that he and M. Garde tried upon their own persons in-
oculation, with the contents of the bullae taken from a case of chronic
pemphigus, " but without other effects than such as follow a simple punc-
ture."
The diagnosis of pemphigus is easy when the bullae are distinct and
unbroken; if they happen to be small and clustered together, the patches
might be mistaken for herpes phlyctenodes, but the bullae are always
larger than the vesicles of herpes, and isolated bullae presenting their
usual characters will be found coexisting. From rupia simplex, pem-
phigus is distinguished by the bullae in the former being smaller, flatter,
and fewer, also by their terminating in ulceration, and in thick promi-
nent scabs. The scabs of pemphigus have been mistaken for those of
ecthyma or impetigo; impetigo, however, is usually confined to a limited
surface, and in ecthyma pustules will be observed in some other part of
the body.
The treatment of acute pemphigus is in general as simple as the dis-
order is mild; laxatives, diluents, and a cooling vegetable diet are usually
alone necessary. In the chronic form of the disease, we must in general
have recourse to tonics early, as wine, quinine, and the mineral acids ;
some prefer the preparations of iron ; Mr. Wilson has seen much benefit
from the hydriodate of potash. As in many cases the affection is com-
plicated with a disordered condition of some one of the abdominal vis-
cera, our treatment must of course be directed to it. The local treat-
ment consists in puncturing the bullae, and gently pressing out the fluid.
" When the bullae have burst, and excoriations remain, anodyne and
emollient fomentations, weakly astringent lotions, or absorbent powders,
(as that of starch,) may be employed with advantage. In these exco-
riations a solution of nitrate of silver, containing two grains to the ounce
will be found the best application. Turner's cerate is also a useful ap-
plication."
Rupia. This affection was classed among vesicular diseases by Willan
and Bateman, but it has been very properly removed to the orders bullae
by Rayer and Biett; it approaches pemphigus in some of its characters,
ecthyma or herpes in others. " It may," says Mr. Wilson, " be regarded
as a modification of pemphigus developed in cachectic and debilitated
constitutionsand according to MM. Cazenave and Schedel, " it
has a great analogy to ecthyma, of which in many cases it appears
to be a variety." Rupia is characterized by small, flattened bullae, which
are usually few in number, and isolated ; filled with a serous fluid which
soon becomes purulent or sanguinolent, succeeded by thick prominent
crusts or scabs, which conceal ulcers of a circular form, and greater or
less extent and depth. The affection is usually chronic, and the lower
extremities are frequently its seat.
Three varieties of rupia are described by most modern writers, viz.
R. simplex, R. prominens, and R. escharotica ; the beautiful engravings
of the two former in Bateman's large work must be familiar to our
readers; the latter has been described under several different names, and
much confusion has been the result. Under the name pemphigus gan-
grenosus, Dr. Whitley Stokes, in the Dublin Medical Essays, has given a
full description of this disease and its treatment; and as it is rather a
1843.] on Diseases of the Skin. 387
frequent disorder in some places, and is often fatal, we propose to devote
a few words to its consideration, more particularly as neither Mr. Wilson
nor Dr. Burgess has alluded to Dr. Stokes's communication.
The disease popularly known in Ireland under the name " burnt holes,"
is not unfrequent in that country among the ill-fed children of the poor.
It has been observed as a sequela of measles, scarlatina, fever, and small-
pox, and in some instances it appears to have prevailed epidemically.
The bullae have an irregularly oblong shape, are flattened at the top, and
the contained fluid soon becomes sanguinolent and finally turbid; they
are surrounded by an inflamed border of a livid red colour; when they
burst, a gangrenous eschar is exposed, or an ulcerated surface which
enlarges rapidly, becomes extremely painful, and is attended with re-
markable fetor, very great discharge, and livid edges. The bullse arise
in succession in different places, and the disease continues with little re-
mission for ten or twelve days, when the patient dies, worn out by pain,
fever, and loss of rest. The disease often makes its first appearance be-
hind the ears, then upon the abdomen, inside of the thighs, &c., and is
very often fatal; its subjects are weakly emaciated children, usually be-
longing to the very poorest class. The treatment of this form of rupia is
of a local and general nature. The most important general measures,
such as change of air, nutritious diet, wine, &c., are too often beyond the
reach of the patient; and quinine or cinchona bark, with the mineral
acids, appear to be less efficacious in this than in other affections ac-
companied by debility. Dr. Stokes recommended the internal admin-
istration of yeast. Anodynes will be almost always found necessary.
Among local applications an ointment, composed principally of the fresh
leaves of the scrophularia nodosa, had long been a popular remedy in
Ireland, and the trials which Dr. Stokes made with it, led him to con-
sider it as much superior to any other. On his recommendation and
authority the ointment was introduced into the Dublin Pharmacopoeia.
He recommends a poultice of oatmeal and porter to be laid on the parts
for a few hours, and the ointment (previously warmed) to be then ap-
plied to the whole ulcerated surface, by means of a feather.
VesiculcB are small elevations of the surface containing a transparent
fluid, formed by the effusion of a little transparent serum under the
cuticle, and differing from bullae merely in size. The fluid is sometimes
reabsorbed, and desquamation takes place, at others the vesicles burst,
and are succeeded by superficial excoriation, or yellow lamellated incrus-
tations, They appear upon every part of the body, are sometimes pre-
ceded by general febrile symptoms, are seldom dangerous and never con-
tagious.
Willan and Bateman comprise seven genera under this order, viz.,
varicella, vaccinia, herpes, rupia, miliaria, eczema, and aphtha; Biett
admits only five, viz., miliaria, varicella, eczema, herpes, and scabies;
Mr. Wilson limits it to three, viz., herpes, eczema, and sudamina. Mr.
Wilson has shown that varicella and vaccinia are variolous affections ;
rupia is generally recognized as a bullous affection; miliaria he treats of
as a consequence of disorder of the sudoriparous system ; and scabies as
an inflammation excited by the presence of parasitic animalcules in-
habiting the epidermis.
Herpss. This term was for a long time employed almost as vaguely
388 Wilson, Cazenave, Schedel, Burgess, Baumes, [April,
as that of" dartre" by the French. Willan first restricted it to a genus
of vesicular diseases characterized by the eruption of clusters of vesicles
upon an inflamed base, which extends a little beyond the margin of
each cluster, seldom attended by much constitutional disturbance,
though of an acute character; usually preceded by heat, tingling
and sometimes pain ; non-contagious; persisting from one week to two
or three, seldom requiring any active treatment, and terminating by ab-
sorption of the contents of the vesicle, " by desiccation without rupture,
or by rupture and the formation of a thin brownish scab, which speedily
falls."
Willan and Bateman describe six species of herpes, and MM. Cazenave
and Schedel follow the same arrangement. Mr. Wilson classes the
species belonging to this genus in two groups, a phlyctenoid and a cir-
cinnate group, an arrangement which appears to be natural.
" The phlyctenoid group is characterized by the irregularity of forin, and dis-
tribution of the clusters of which it is composed; it is typified by the variety
herpes phlyctenodes, and embraces all the local forms. The circinnate group
on the other hand is remarkable for the circular arrangement or form of its
clusters: hence the herpes zoster consists of irregular clusters disposed in a
circular form round the trunk; herpes circinnatus (vesicular ringworm) is
characterized by the disposition of individual vesicles in the form of a circle ;
and herpes iris presents the same peculiarity in the form of concentric circles."
(p. 149.)
Suppurative inflammation of the dermis. Under this title Mr.
Wilson includes the "pustular diseases of the skin," but he limits consi-
derably the number of genera included in this order by other writers.
Pustules are small circumscribed elevations, formed by the effusion of a
purulent fluid between the cuticle and dermis. " Vesicles, it is true,
at a certain period, contain a thick sero-purulent fluid, but it is alto-
gether consecutive of the transparent serous fluid; while the pustular
eruptions contain true pus from the beginning; besides the physical cha-
racter of this fluid, which is thick and yellow, will readily distinguish it
from the opaque coloured serum which vesicles contain just before their
disappearance." In addition, pustules usually differ from vesicles in the
depth and intensity of the inflammation which accompanies them.
Pustules were reduced by Willan to two principal or essential forms,
viz. phlyzacia and psydracia. The phlyzacious pustules (derived from
<p\v?eiv, to be hot,) are of a large size, have a highly inflamed base, as
the name indicates, and are succeeded by hard, thick, dark-coloured
scabs, as seen in ecthyma. The psydracious pustules (quasi ^vyjpa vbpaicia,
i. e. frigid? guttulae,) are of smaller size, " characterized by a less
degree of surrounding inflammation," and by terminating in laminated
scabs ; they are seen in impetigo.
The order Pustulae in Willan's arrangement includes five diseases, viz.
Impetigo, Porrigo, Ecthyma, Variola, and Scabies. M. Biett's classifi-
cation embraces seven, viz. Variola, Vaccinia, Ecthyma, Impetigo, Acne,
Mentagra, and Porrigo, to which Dr. Burgess has added an eighth,
Equinia, or glanders. M. Rayer admits as many as ten genera into his
order pustulse. Mr. Wilson considers only two diseases under this head,
Impetigo and Ecthyma; he treats of acne and sycosis under "diseases of
the sebaceous glands;" favus (the true porrigo of other writers) under
" diseases of the hairs and hair follicles;" and variola, vaccinia, and va-
1843.] on Diseases of the Skin. 389
ticella under " congestive inflammation of the dermis." In impetigo and
ecthyma we have examples of psydracious and phlyzacious pustules, the
former are seen in impetigo, the latter in ecthyma.
Impetigo. This disease is characterized by an eruption of small flat-
tened pustules without an inflamed areola, unaccompanied by fever, and
non-contagious; " each pustule attains its full development, and bursts
in the course of two or three days, terminating in rough, yellowish, semi-
transparent crusts, of considerable thickness. The disease occurs in suc-
cessive crops, is attended with trifling or no constitutional symptoms, and
endures for three or four weeks to as many months or even years." The
pustules of impetigo are sometimes collected into clusters, (impetigo
figurata Willan;) in others they are more or less scattered, (I. sparsa
Willan.) To these two varieties, or a combination of them, the several
forms of impetigo described by Willan may be referred, with the exception
of I. rodens, " which is rather a cancerous affection than a species of im-
petigo," and the porrigo larvalis and P. granulata of Willan are now
known to be forms of impetigo, and were first described as such by Biett,
under the names impetigo larvalis and I. granulata.
Diagnosis. Impetigo of the scalp is sometimes confounded with favus
or eczema impetigenodes of this region. From favus it is distinguished
by the pustules in the latter being deeply seated in the substance of the
skin, and by their rapidly changing into dry yellowish cup-shaped scabs;
in addition, favus is a contagious disease, and implicates the hair-bulbs,
causing the falling off of the hair, the contrary of which is observed in
impetigo. From eczema impetigenodes it is distinguished by the presence
of vesicles in the latter, as well as by the form of the crusts or scabs,
which are thin and lamellated. Impetigo affecting the chin only has
been mistaken for sycosis; the pustules of sycosis, however, are larger,
more raised, less yellow than those of impetigo, and are accompanied by
less secretion ; " the crusts of sycosis are darker in colour, not renewed
when they fall off, and accompanied by tubercles and indurations."
Bateman observes that impetigo in its advanced stages is liable to be
mistaken for psoriasis or lepra, " and is in fact daily mistaken for these
diseases;" but a practitioner who could commit such an error of diag-
nosis must be entirely unacquainted with the characters of the two dis-
eases; the scaly diseases emit no fluid, and "the very existence of a
discharge, however slight, is sufficient to determine the point."
Treatment. We agree with MM. Cazenave and Schedel that the
preparations of sulphur have been too generally recommended in impe-
tigo, and that their indiscriminate employment, especially in the early
stage, is often decidedly injurious. When the disease is limited, and the
local symptoms are mild, emollient and sedative fomentations, the warm
bath, vapour bath, or douche, are the best local applications that we can
employ, acidulated drinks being at the same time administered internally :
general or local bleeding is seldom necessary. " In the treatment of
impetigo," Mr. Plumbe well observes, " the frequent removal of the dis-
eased secretion has never been considered of sufficient importance ; the
benefit of this step, if carried into effect by frequent ablution of the part
with warm water, is incalculable. By this plan, in conjunction with the
exhibition of simple alteratives, entirely rejecting anything in the shape
of ointments, or other greasy applications, the disease will be often readily
390 Wilson, Cazenave, Schedel, Burgess, Baumes, [April,
subdued. The part may be kept in a state of moisture at other times,
by covering it with oilskin, or by the application of soft linen wetted with
the Liq. pluinbi acet. dil." The hydrocyanic acid in the form of lotion,
combined with alcohol and acetate of lead has been highly recommended
by Dr. A. T. Thomson; he observes that he found it to allay the irrita-
tion more effectually than any other application; the following is the
formula which he employs :
R Acidi hydrocyanici . f. 3 iv.
Aqufe distillatae . f. 3 vij.
Alcoholis . f. 3 iv.
Acetatis plumbi . gr. xvij.
M. ft. Lotio.
Mr. Plumbe, in his Practical Treatise, recommends a formula nearly
similar, but without the acetate of lead, and observes that " of all external
applications he has found none equal in efficacy to it;" he directs the
part to be kept constantly covered with pieces of linen wetted with it.
M. Rayer, however, observes that lotions composed of sulphuric or nitric
acid largely diluted with water are equally efficacious.
In the chronic forms of impetigo, the sulphureous preparations are
more decidedly indicated, and may be employed both internally and ex-
ternally ; and the warm bath and vapour douche here will be found valu-
able adjuncts in restoring the healthy action of the skin. " Should the
disease resist these measures, lotions containing sulphuret of potash, ni-
tric acid, or nitrate of silver, may be in turn had recourse to." Creosote
ointment, oxide of zinc, acetate of lead, and citrine ointment have been
also employed with advantage; and when the disease is limited, a blister
applied to the diseased part, as recommended by MM. Cazenave and
Schedel, has often proved beneficial. M. Rayer says he has treated very
obstinate impetigo successfully by nitric acid, in doses of half a drachm
daily in a pint of barley-water, sweetened to the taste; " it very seldom
happens that this medicine is continued for a month or six weeks without
accomplishing a cure." If all these remedies fail, MM. Cazenave and
Schedel observe, we must have recourse to the arsenical preparations,
(as Fowler's solution,) which is generally followed by the most surprising
effects."
" In impetigo of the hairy scalp, the hair should be cropped over the diseased
parts, and the crusts completely removed by means of the vapour douche and
water dressings. The parts should be kept free from the irritation of fresh in-
crustations by frequent washing, and the same remedial measures pursued, as
recommended for impetigo in other parts of the body." (Wilson, p. 1870
Artificial pustular eruptions. It is well known that the application
of some inorganic substances to the surface, particularly tartar-emetic, is
capable of producing the development of a crop of pustules at the part,
resembling in some respects those of variola; and artificial eruptions by
means of this substance are usefully excited in a great number of different
diseases. The rapidity with which the pustules are developed will de-
pend upon the form in which the tartar-emetic is applied, the fineness or
irritability of the skin, &c. It is commonly applied in the form of oint-
ment, and the active ingredient of the ointment contained in the phar-
macopoeia may be advantageously increased or lessened according to cir-
1843.] on Diseases of the Skin. 391
curastances. Another method is to sprinkle the powder upon soap-
plaster, common adhesive, or Burgundy pitch plaster, and allow it to
remain upon the part, by which larger and more painful pustules are ge-
nerally produced than by the ointment. Dr. Lichtenstein (in a paper
quoted by Mr. Wilson,) remarks that limpid lymph taken from the pus-
tules produced by tartar-emetic, and inoculated in a person who has not
been vaccinated, produces vesicles which cannot be distinguished from
those of vaccinia; and which appear to be equally protective against
smallpox with the cowpox; the matter may also be transmitted from
person to person in the same manner. The author has inoculated and
reinoculated thirty-one persons with the matter procured from this source,
and they were protected during an epidemic of smallpox, although placed
in association with patients affected with that disease.
Pustules from the inoculation of animal matters. Various animal
matters, when applied to the surface for a longer or shorter period, or di-
rectly introduced by means of inoculation, are capable also, as is well
known, of causing the development of pustules. Variola and syphilis
are familiar examples; dissecting wounds are also frequently followed by
pustules; and the morbid matter secreted in horses labouring under
glanders or farcy may, it is now well known, be communicated to the
human subject, and give rise to a very fatal disease. Dr. Burgess, in
his translation of MM. Cazenave and Schedel's work, has added to the
chapter on pustular diseases an excellent account of glanders and farcy
as they occur in the human subject.
Had we not in a recent Number, (vol. XIII. p. 28,) given so full an
account of these diseases, both in animals and man, we should have
gladly extracted Dr. Burgess's abstract. The reader will find the details
very interesting.
Depositive inflammation of the dermis. Under this title, (se-
lected, he says, only in the absence of a more suitable one,) Mr. Wilson
includes diseases characterized by the effusion of plastic lymph into the
tissue of one or more of the papillae of the dermis, constituting a pimple
of small size. Mr. Plumbe regards a pimple as formed not by the en-
largement of a papilla, but by the escape of a minute quantity of lymph
from a distended vessel. This division includes the papular diseases of the
skin of other writers, the principal of which are lichen and prurigo.
A papula or pimple is a small, solid, hard, acuminated elevation of the
dermis, attended with constant and distressing itching. The number of
diseases admitted into this order by Willan was three; Mr. Wilson also
admits three, viz. Strophulus, Lichen, and Prurigo; Biett, Rayer, and
Gibert reduce the number to two, considering strophulus to be merely a
modification of lichen peculiar to newly-born or sucking infants; and
Alibert considers them all under a single genus, Prurigo. The diseases
included in this order are seldom attended by febrile symptoms, and
are usually of a chronic nature ; they are non-contagious; none of them
are dangerous, and their diagnosis is usually exceedingly easy. The
most prominent symptom which accompanies them is itching; they ter-
minate in resolution or more frequently in desquamation.
Strophulus. Mr. Wilson follows Willan and Bateman in making five
species of this cutaneous eruption, the distinctions being founded on the
appearance, distribution, and colour of the pimples; these are all in-
392 Wilson, Cazenave, Sciiedel, Burgess, Baumes, [April,
eluded by Biett under one name, viz. lichen strophulus. The eruptions
referred to the genus strophulus are " better known to mothers and
nurses than to the physician," by whom they are variously named tooth-
rash, gum, red or white gum ; they are peculiar to infants at the breast,
and are often associated with dentition or gastro-intestinal irritation ;
they are wholly unattended by danger.
Lichen is characterized by an eruption of minute conical papulae, which
are hard, slightly red, or of the natural colour of the skin, usually occur-
ring in clusters, and appearing in single or successive eruptions, occu-
pying a particular region or disseminated over the whole surface; their
size is about that of millet seeds, and they are attended by a sensation of
tingling or itching; lichen is sometimes ushered in by febrile symptoms,
runs an acute course, and ends in two or three weeks; more frequently
it is of a chronic character, and may persist for months or years.
Lichen differs from strophulus by occurring in the adult; it selects in
preference those parts of the surface in which the dermis is thickest, as
the back, outer side of the limbs, face, &c. It appears under several
different forms, eight varieties (which have received distinct names) being
noticed by Mr. Wilson. It would far exceed our limits to enter mi-
nutely upon the consideration of the characters of the different varieties
of lichen ; the following animated description of Lichen tropicus, or
prickly heat, (given by Dr. James Johnson, in his work on Tropical Cli-
mates, from which he was himself a sufferer, and quoted by Mr. Wilson,)
may prove interesting:
" The sensations arising from prickly heat are perfectly indescribable, being
compounded of pricking, itching, tingling, and many other feelings for which I
have no appropriate appellation. It is usually, but not invariably, accompanied
by an eruption of vivid red pimples, not larger in general than a pin's head,
which spread over the breast, arms, thighs, neck, and occasionally along the
forehead. This eruption often disappears in a great measure when we are sit-
ting quiet, and the skin is cool; but no sooner do we use any exercise that
brings out perspiration, or swallow any warm or stimulating fluid, as tea, soup,
or wine, than the pimples become elevated, so as to be distinctly seen and but
too sensibly felt. This unwelcome guest assails us at all and particularly the
most unseasonable hours. Many a time have I been forced to spring from
table and abandon the repast which I had scarcely touched, to writhe about in the
open air for a quarter of an hour; and often have I returned to the charge with
no better success against my ignoble opponent. The night affords no asylum.
For some weeks after arriving in India, I seldom could obtain more than an
hour's sleep at one time, before I was compelled to quit my couch with no
small precipitation, and if there was any water at hand, to sluice it over me,
for the purpose of allaying the inexpressible irritation. But this was produc-
tive of temporary relief only, and what was worse, a more violent paroxysm fre-
quently succeeded." (p. 202 )
Prurigo. Amongst papular diseases prurigo is perhaps the most for-
midable, and certainly the most intractable which we meet in this
country; the variety termed Prurigo senilis is the most frequent, and the
difficulty of its cure is almost proverbial. It occurs commonly in per-
sons advanced in life, whose constitutions have been debilitated from
any cause; it is more frequently seen in humble life than in the affluent,
and we have observed it several times in individuals who had suffered a
reverse of fortune, and from easy circumstances had been exposed to all
1843.] on Diseases of the Skin. 393
the privations of poverty. The disease is so well known that we need
not delay to dwell upon its characters or symptoms, but shall confine our
observations merely to its treatment.
The treatment of prurigo senilis as laid down in books is for the most
part palliative, and is directed either to the alleviation of the itching,
(the most troublesome symptom,) or to destroy the pediculi which some-
times accompany this cutaneous eruption ; as tepid baths of salt or fresh
water, sulphurous baths, lotions containing acetic acid, corrosive subli-
mate, sulphuret of potash, muriate of ammonia, &c.; or lime-water or
spirits of turpentine mixed with almond oil; inunction with mercurial
ointment or an ointment containing white hellebore (veratrum album),
or muriate of ammonia, or fumigations with cinnabar. Many authors
indeed set down this disease as incurable, or nearly so : " the frequent
occurrence of this disease in old age, and the difficulty of curing it,"
Bateman observes, " have been the subject of universal observation."
" Prurigo senilis," says Rayer, " sometimes resists the best combined
methods of treatment;" and Biett observes, "this disease is formidable
for its obstinacy, and is sometimes incurable." In some instances iodine
locally applied has been found serviceable; Mr. Wilson says " he has
used this remedy both as a local and general remedy with good results."
" Observing the serious effects that sometimes result from the retrocession
of prurigo, I was induced," Mr. Wilson remarks, " to make trial in ob-
stinate cases of a stimulating liniment applied to the skin, after a previous
course of tepid baths, and with the most beneficial results. The appli-
cation which I employ in such cases is croton oil, diluted with oil of
almonds, in the proportion of half a drachm or a drachm of the ounce."
Mr. Plumbe refers to a very obstinate case of prurigo cured by the fol-
lowing remedies : the papulae were first touched with lint dipped in undi-
luted aromatic vinegar, which caused a good deal of pain, the following
ointment was then employed :
Sulphur sublim.
Picis liquidae.
Axung. porcin. aa lb. ss.
Terrae cretos. v.
Hydrosulphuret. auamoniae 3ij.
it linemen film.
M. Fiat unguentum
In three days the acid was again applied, and afterwards a solution of
nitrate of silver every third or fourth day, previous to the application of
the ointment. The internal treatment consisted in the administration of
four grains of Plummer's pill at night, and five drops of the arsenical
solution three times a day. In less than three weeks the eruption had
nearly disappeared, and the itching was entirely removed; the pills and
solution were continued for three weeks longer, during which time a
lotion of corrosive sublimate in spirits of wine was applied two or three
times a day, instead of the ointment. M. Biett employs the ioduret of
sulphur in the form of ointment in prurigo senilis; the following is his
formula:
ft. Ioduret. sulphuris, gr. xx-xxx.
Axung. unciam.
M. Fiat unguentum.
394 Wilson, Cazenave, Schedel, Burgess, Baumes, [April,
Alibert recommended the following ointment in this disease :
R. Tinct. opii.
Sulphur, sublim. aa Jss.
Oxidi zinc.
01. arnygdal. aa ^j.
Adipis. |iij. M.
We have derived more benefit from the local application of creosote in
this troublesome affection than from any other remedy. The creosote may
be employed in the form of ointment or lotion or both combined. The
proportion of creosote contained in the ointment is from eight to sixteen
drops to the ounce of lard ; in the lotion half a drachm of creosote to
eight ounces of water, to which a little acetic acid or spirits of wine is
added to render it soluble in the water. The ointment should be freely
rubbed upon the parts at night, and the lotion applied the following
morning, and repeated during the day; the itching is very speedily re-
moved, and the disease by perseverance in the remedies for a short time
subsides; the previous employment of the warm bath is generally advi-
sable, and in all cases the linen should be frequently changed. At the
same time the general health must be attended to; the occasional ad-
ministration of a combination of purgatives with a tonic will be found
useful; all stimulating articles of food must be prohibited.
Squamous inflammation of the dermis. Under this designation
Mr. Wilson includes a class of diseases characterized by inflammation of
the dermis, and by the production of abnormal epidermis in the form of
thin laminae or scabs : these are the scaly diseases of other writers, and
they belong to the division of Mr. Plumbe, entitled " diseases chiefly
marked by chronic inflammation of the vessels secreting the cuticle, pro-
ducing morbid growth of this structure."
The scaly diseases of the skin are characterized by the formation of
inorganic laminated scales of morbid cuticle (squamce), of a whitish
colour, opaque, dry, friable, and more or less adherent; they are elevated
above the rest of the skin, and then thrown off; the skin appears red and
inflamed, and they are speedily reproduced. The eruption commences
with red, slightly elevated, and distinct spots or patches, which some-
times remain distinct, at others coalesce, and upon which squamse are
soon formed. The commencement of the eruption " is rarely attended
with constitutional disturbance; indeed, the patient is frequently not
aware of the existence of the disease until the patches are fully formed,
or the cuticle is on the point of being detached." The only consti-
tutional symptoms which accompany them are a slight degree of heat and
itching ; they are not contagious, occur generally in adults, and are more
frequently seen in the female than the male; they are not dangerous,
but are usually exceedingly tedious and difficult to cure. The diagnosis
of the scaly diseases of the skin is generally easy, the laminated scales
which they present are sufficient to distinguish them from every other
cutaneous eruption; in addition the scaly diseases (as remarked by
Bateman,) emit no fluid, and the presence of a discharge, however slight,
is sufficient to determine the point.
The order Squamse in Willan's arrangement includes four diseases,
viz., lepra, psoriasis, pityriasis, and icthyosis; the latter which bears no
analogy to the others has been very properly removed from this order by
1843.] on Diseases of the Skin. 395
Rayer and Mr. Wilson, although still associated with it by Biett and
MM. Cazenave and Schedel.
Lepra and psoriasis are the type of the scaly diseases of the skin :
both commence in small papular looking elevations of the surface, which
soon become covered with a laminated scale, of a white silvery appear-
ance. " These elevations unite, and then change into scaly patches, of
various forms and dimensions, which may either be in small numbers,
and limited to a single region of the body, or occur disseminated over
the whole surface. In the latter case the desquamation is frequently so
copious that the bed and clothes of the patient become filled with dry
whitish-looking scales." The eruption most frequently commences on
the extremities, particularly the neighbourhood of the knee and elbow,
from which it extends to the body generally. It is usually stated that
the scalp is not often attacked ; but when the eruption is general over the
body, we have found the scalp to be more or less engaged. Lepra and
psoriasis have so many characters in common, and so much analogy with
each other, that several writers upon cutaneous diseases include both
under one head, considering them to be merely varieties of the same af-
fection ; and we have no doubt this is the correct view to take ; for in-
stance, the two diseases not unfrequently complicate one another; we
have seen them combined, and again passing into each other, so as to
lead to considerable difficulty in the diagnosis. We seldom or never
find them combined with other forms of cutaneous eruptions ; they are
both of a chronic nature, rebellious to treatment, and often very difficult
to cure; and lastly the treatment found to be effectual in relieving the
one is usually successful also against the other.
" If any useful purpose were to be gained by their reunion," Mr. Wilson
observes, " I would cheerfully record my vote in its favour, for the similarity
of lepra and psoriasis in their essential nature is so complete, as to render them
almost identical. On the other hand, it maybe fairly advanced, that the terms
are so well understood, that no error can arise out of their separate existence;
that time has rendered them classic sounds, which could not well be dispensed
with; and moreover that certain differences of moment are admitted between
them, such as extent of surface occupied, duration and severity." (p. 213.)
Treatment. The treatment of lepra and psoriasis is essentially the
same ; the latter disease is however in general the most obstinate ; both
local and constitutional remedies are usually necessary, the former when
employed alone, always (as Biett observes,) proving ineffectual, and often
not only useless but injurious. The majority of cases of these diseases
are unattended at any period by active inflammation or much irritability of
the surface, hence it is seldom necessary to resort to bloodletting, or the
antiphlogistic regimen; though in recent cases, and " where the disease
pursues an acute course" MM. Cazenave and Schedel observe they may
be required ; such cases, however, must be very rare.
The local remedies are warm sulphureous or salt-water baths, and the
vapour bath, which, according to M. Biett, excels all the others; sulphur
fumigations are more efficacious in psoriasis than lepra; towards the de-
cline of the eruption an ointment of the ioduret of sulphur, in the pro-
portion of twenty or thirty grains to the ounce of lard, is frequently used
at the hospital of St. Louis with the best effects. M. Gibert employs an
ointment of fuligokali (a compound of soot and potash) in the proportion
396 Wilson, Cazenave, Schedel, Burgess, Baumjes, [April,
of twenty or thirty grains to the ounce of lard ; and Mr. Wilson says he
has used this substance in local psoriasis with better success than he had
obtained by the usual remedies. M. Lemery applies naphthaline in the
form of ointment to the diseased surface on folds of linen : his ointment
consists of two to four parts naphthaline to thirty parts of lard ; several
other forms of ointment are also occasionally resorted to, and the white
precipitate, oxide of zinc, or citrine ointment. Mr. Plumbe states that
lie has derived much benefit from the following ointment in lepra, lupus,
and several other obstinate cutaneous affections; " its composition," he
adds, " may not perhaps bear the test of chemical criticism, but as reme-
dies are estimated by their useful effects, this is of no importance
R Hydrargyri submuriatis.
Plumbi superacetatis - aa 3ss
Unguent, hydrarg. nit.
cetacei - - aa gij
M. Fiat ungucntuin.
External remedies alone, as already observed, are not competent to
remove these diseases, and ought not to be persevered in too long. The
internal remedies which have been recommended in these diseases are very
numerous, but, as Bateman observes, " there is no one remedy, nor any
invariable plan of treatment which will succeed in every instance, and
under all circumstances." The remedies advocated by the highest au-
thorities, and whose value has been most frequently tested, are the pre-
parations of arsenic, mercury, and iodine, or combinations of them ;
tincture of cantharides, dulcamara, sarsaparilla, and purgatives. " The
result of M. Biett's experience at the Hospital of St. Louis goes to prove
that the most successful internal remedy consists in the exhibition of pur-
gatives, tincture of cantharides, and the different preparations of arsenic."
Purgatives are only serviceable when the disease is recent, of limited ex-
tent, and affects young persons. " The tincture of cantharides adminis-
tered internally in some mucilaginous drink, in doses gradually increased
from five to twenty or thirty drops several times a day, occasionally," says
Rayer, 44 causes the rapid disappearance of the disease, especially when it
is limited, and not very severe in its character." MM. Cazenave and
Schedel consider this medicine more useful in cases where the disease has
reappeared, or is diffused, or where it has resisted the action of purgatives,
and occurs in subjects of a soft and lymphatic constitution ; " it gene-
rally effects a cure in the course of forty-five to fifty days, especially in
females." They mention a case of lepra of eighteen years' standing,
treated at St. Louis, by this medicine, in which the eruption disappeared
in the course of a month. The objections to the employment of tincture
of cantharides are its liability to excite strangury, and its tendency to
derange the digestive organs ; on which account we are often obliged to
intermit its use, or sometimes to give it up altogether: it does not appear
to be quite so effectual in psoriasis as in lepra, and it usually succeeds
better in the cases of females than in males.
The preparation of arsenic ordinarily employed, and perhaps the best,
is Fowler's solution. We may commence with a dose of two or three
drops twice or three times a day, and gradually and cautiously increase
it to ten or twelve drops ; it may be administered in a decoction of dul-
camara, sarsaparilla, or cinchona; and it must be persevered in regu-
1843.] on Diseases of the Skin. 397
larly, frequently for two or three months; it often at first increases the
redness and heat; and its effects require to be carefully watched; if it
produces a sensation of stiffness or tension of the eyelids, pricking in the
throat, puffiness of the face, thirst, redness of the tip and edges of the
tongue, or pain at the epigastrium, it must be immediately discontinued.
Dr. A. T. Thomson prefers the iodide of arsenic to Fowler's solution, in
doses of one tenth of a grain ; he also recommends the biniodide of mer-
cury, in combination with opium or conium, in doses of from one sixth
to one fourth of a grain, and in obstinate cases he combines it with the
iodide of arsenic. More recently Mr. Donovan of Dublin has introduced
a combination of arsenic, iodine, and mercury, to the notice of the pro-
fession, under the name " Liquor hydriodatis arsenici et hydrargyri,"
which has been found very serviceable in cases of psoriasis and lepra; each
drachm of this solution contains one eighth of a grain of protoxide of ar-
senic, one fourth of a grain of protoxide of mercury, and four fifths of a
grain of iodine converted into hydriodic acid. The dose is half a fluid
drachm three times a day, for an adult. Several other remedies have been
recommended in these diseases : Biett states he has not found " the decoc-
tion of dulcamara of much use ; tar and pitch have invariably failed with
him ; and sarsaparilla, mezereon, and white hellebore are very uncertain."
Inflammation of the dermis induced by parasitic animalcules
inhabiting the epidermis. This is the title of the eighth chapter of
Mr. Wilson's work. It contains but a single disease, scabies, which by
most writers is referred to the order vesiculae, and by Willan was erro-
neously placed among pustular diseases. Mr. Wilson looks upon the
presence of the acarus scabiei as a necessary feature in the diagnosis of
the disease.
" When one of the early vesicles of scabies is examined with attention, a mi-
nute spot or streak may be observed upon some one point of its surface. This
is the aperture originally made by the insect on its first entrance beneath the
epidermis, and from this spot or streak a whitish line may be traced, either in
a straight or curved direction, into the neighbouring epidermis. The whitish
line is the cuniculus or burrow of the acarus; it necessarily varies in length,
being sometimes as much as five or six lines in extent, and at its termination,
under a slight elevation of the epidermis, the little inhabitant lies concealed.
The acarus may be easily distinguished by the experienced eye as a small dark
point at the end of the cuniculus, and if a thin capsule of epidermis be raised
in this situation with the point of a needle, the little creature is brought into
view. . . . There is no communication between the vesicle and the cuni-
culus, and the acarus is never situated within the vesicle or pustule." (p. 239.)
" The existence of the acarus scabiei is without question; I have extracted as
many as twenty at a single sitting. I have placed them on a slide of glass and
seen them run; and after the business of the day has been over, I have ex-
amined them with the microscope, and found them still active. When examined
with the naked eye, the acarus looks white and shining, globular in its form,
and very aptly resembling the little bladder of water of bonomo. There is no
difficulty in extracting it; the cuniculus is seen without difficulty; the end
of the cuniculus is perceived to be a little raised; as soon as this little eminence
of epidermis is lifted, ifjthe end of the needle or pin be examined, the minute,
white, shining globe will probably be observed attached to the instrument.
This facility of extracting the animal is due, in a great measure, to
its power of clinging by means of a special apparatus which it possesses."
(p. 381 et seq.)
Mr. Wilson regards the acarus scabiei as " the unique cause of scabies,
VOL. XV. NU. XXX. '8
398 Wilson, Cazenave, &c. on Diseases of the Skiyi. [April,
and as a necessary feature in the diagnosis of the disease." The animal
is transferred by the infected to those who are sound by actual contact;
in some cases the adult acarus, in others " ova or embryos suspended in
the fluid of the vesicles may be the mode of transmission." " Certain it
is," he adds, " the application of one of these animalcules to the skin of
a sound person will give rise to the disease." Mr. Wilson then relates a
series of experiments performed by Mr. Albin Gras, a pupil at St. Louis,
published by that gentleman in a short memoir on the subject, in the
year 1834, two of which we give in the author's words.
"I lately placed nine acari in the bend of my left arm, and retained them
there by a compress and bandage. Four hours after, I felt considerable pru-
ritus, and next day perceived four cuniculi. Several days after, some vesicles
showed themselves on my forearm. Having placed two acari in the flexure of
the elbow of two persons, who expressed their willingness to submit to my ex-
periments; on one, three or four vesicles were apparent on the fifth day; and
were accompanied by severe itching. On the other, there were two cuniculi,
with pruritus, but no vesicles." (p. 224.)
In connexion with parasitic animalcules, we may here allude to another
which has been recently found to inhabit the sebaceous follicles of which
Mr. Wilson has given a figure and description. In his researches to
discover the cause of acne, Dr. Simon, of Berlin, made the discovery of
this animal, to which he has given the name " acarus folliculorumit
is found imbedded in the sebaceous matter near the orifice of the follicle,
the head being directed inwards. These animalcules are tardy in their
movements, and retain their vitality for a considerable time: thus M.
Simon found them alive after ten or twelve hours' confinement between
two plates of glass; and in one instance they were found alive in a sub-
ject dead for six days.
"After perusing the account of the acarus folliculorum given by Dr. Simon,
I determined (says Mr. Wilson,) to proceed to a verification of his discoveries.
I was not long in obtaining subjects, almost every face that I met supplied me
with abundance; and the difficulty seems to be, not to find the creature, but to
find any individual, with the exception of newly-born children, in whom they do
not exist almost every collection of sebaceous matter which can be squeezed
from the numberless cutaneous apertures upon the nose, forehead, face, &c.,
will furnish subjects, and as the parasites are situated near the mouth of the
follicle, that portion which is squeezed out with the least force is the part most
likely to be inhabited by the acarus. The acarus folliculorum would seem to
give rise to no uncomfortable effects by its presence, unless perchance it should
multiply to such an extent as to become a source of irritation to the follicle...
. .These animalcules undoubtedly feed on the sebaceous substance in which they
lie imbedded, and which is the cause of their existence. I have commonly
found two in the small mass of this substance expressed by the fingers, often
four and five, and in one instance eight closely connected together." (p. 389.)
With these quotations our limits oblige us to conclude the notice of
the volumes before us. Much important matter still remains to which
we would gladly have referred, particularly the chapters on syphilitic
eruptions, molluscum, fctvus, acne, &c. In our observation we have been
constrained by the great variety and extent of the subjects to make our
review rather an abstract of the most important points connected with
the diagnosis and treatment of a few diseases ; and our opinion of the
comparative merits of the three treatises may be gathered in some
measure from the number of references we have made to the opinions ad-
vanced or to the descriptions given in each work.
1843.] Kerst and Gobee on Clinical Surgery and Medicine. 399
Mr. Wilson's volume contains an excellent digest of the actual amount
of knowledge of cutaneous diseases; it includes almost every fact or
opinion of importance connected with the anatomy and pathology of the
skin ; and, taken as a whole, is very creditable to him. Of the Manual
of Diseases of the Skin, translated by Dr. Burgess, it is hardly necessary
to give an opinion ; the original has been in the hands of the profession
for several years, and has received the most unequivocal stamp of its ap-
probation, by several editions having been called for during that period.
We shall only add, that the translation is well and faithfully executed,
and the additional matter supplied by Dr. Burgess increases considerably
its utility. M. Baumes' treatise, though containing more than double
the number of pages of either of the former works, has little to recom-
mend it; its arrangement (as we have already observed) is faulty; the
style is too diffuse; and the facts and observations of value which it ne-
cessarily contains, are so mixed up with and obscured by his theory, that,
in the majority of instances, it is difficult to arrive at tHfcm.

				

